# Advancing Osteoporosis Evaluation Procedures: Detailed Computational Analysis of Regional Structural Vulnerabilities in Osteoporotic Bone

**DOI:** 10.3390/jpm13020321

**Published:** 2023-02-13

**Authors:** Matthew A. Wysocki, Scott T. Doyle

**Affiliations:** Department of Pathology and Anatomical Sciences, Jacobs School of Medicine and Biomedical Sciences, University at Buffalo, Buffalo, NY 14260, USA

**Keywords:** biomechanics, computational analysis, diagnostic methods, fracture prevention, personalized medicine, patient-specific

## Abstract

Osteoporotic fractures of the femur are associated with poor healing, disability, reduced quality of life, and high mortality rates within 1 year. Moreover, osteoporotic fractures of the femur are still considered to be an unsolved problem in orthopedic surgery. In order to more effectively identify osteoporosis-related fracture risk and develop advanced treatment approaches for femur fractures, it is necessary to acquire a greater understanding of how osteoporosis alters the diaphyseal structure and biomechanical characteristics. The current investigation uses computational analyses to comprehensively examine how femur structure and its associated properties differ between healthy and osteoporotic bones. The results indicate statistically significant differences in multiple geometric properties between healthy femurs and osteoporotic femurs. Additionally, localized disparities in the geometric properties are evident. Overall, this approach will be beneficial in the development of new diagnostic procedures for highly detailed patient-specific detection of fracture risk, for establishing novel injury prevention treatments, and for informing advanced surgical solutions.

## 1. Introduction

Osteoporotic fractures of the femur are a common injury that results in severe health consequences, including high risk of mortality, disability, and dramatically reduced quality of life [1,2]. Much of the previous research has focused on the relationship between osteoporosis and fractures in the proximal femur because this type of fracture is associated with high mortality rates (i.e., 8 percent within 30 days and 25 percent within 12 months) [3,4,5,6,7]. Although proximal femur fractures have been more frequently studied, it is still critical to study diaphyseal and distal femur fractures because they are also associated with high mortality rates. Diaphyseal femur fractures have a 30 day mortality rate of 6 percent and a 12 month mortality rate of 18 percent, and distal femur fractures have a 30 day mortality rate of 5 percent and a 12 month mortality rate of 18 percent [3]. Patients 80 years and older who have diaphyseal femur fractures have a mortality rate of 28 percent within 12 months [8]. Similarly, elderly patients with distal femur fractures have 12 month mortality rates of 30 percent or greater [3,9,10]. Given the high levels of mortality, the high levels of disability, and the considerable burden placed on the healthcare system, it is essential to advance the understanding of how osteoporosis influences the femur’s structure [1,9,10,11].

Aside from the immediate negative consequences of osteoporotic femur fractures, this injury often has very poor patient outcomes regarding healing, restoration of mobility, and restoration of independent living [1,11]. Osteoporosis-associated femur fractures are still considered to be an unsolved problem in orthopedic surgery because it is extremely challenging to acquire effective implant anchorage and because advanced surgical skills are required for administering treatment [9,12]. Clinical research has shown that it is essential for patients to immediately have their mobility restored following osteoporotic bone fractures. However, fracture fixation frequently fails because of the increased fragility of the osteoporotic bone and the decreased structural capacity of osteoporotic bone to securely hold a surgical implant [13].

Individuals afflicted with generalized osteoporosis are at substantial risk of femur fractures, but most fractures of the femur actually occur in individuals who do not exhibit widespread osteoporosis [14,15]. There is evidence to suggest that most fractures of the femur begin within the cortical bone [4,5,16,17]. In particular, results indicate that proximal femur fractures in females often occur due to localized morphological changes caused by osteoporosis [18]. Considering that small structural failures may be the cause of catastrophic fractures, clinically relevant research has shifted to understanding specific aspects of the femur’s structure rather than the overall bone density of the femur [19]. However, a more comprehensive understanding of how the process of osteoporosis influences the localized morphological and biomechanical attributes of the rest of the femur is still needed.

Computational analysis of the geometric properties of an osteoporotic femur’s structure has the potential to reveal key insights about morphological and biomechanical attributes at specific locations in the femur. The use of the geometric properties from long bones to study biomechanics and functional morphology originated from anthropological studies that employed manual data collection techniques [20]. Over the decades, these underlying principles have been combined with sophisticated digital imaging and 3D modeling techniques to permit improved data collection and data analysis [21,22]. This approach has been used to support the study of the evolution, locomotor capabilities, and behavior of humans as well as other species [23,24,25]. These computational evaluations have also been used to detect changes in healthy long bones due to distinct habitual loading (i.e., bone functional adaptation) patterns, such as the identification of differences in the lower limb bone characteristics of athletes from various sports [26,27,28,29].

In order to more effectively identify osteoporosis-related fracture risk and develop advanced treatment approaches for femur fractures, it is necessary to acquire a greater understanding of how osteoporosis alters the femur’s structure and biomechanical characteristics. The current investigation uses computational analyses to comprehensively examine the specific ways in which the femur’s structure and its geometric properties differ at specific sites across healthy and osteoporotic femurs. It is hypothesized that healthy and osteoporotic femurs exhibit localized disparities (as opposed to uniform differences across the diaphysis) in their geometric properties.

## 2. Materials and Methods

### 2.1. Anatomical Data

We obtained 3D anatomical models of the left femur’s osteological structure (*n* = 42) from cadaveric computed tomography (CT) data and used them to calculate the geometric properties. These CT data were made possible by charitable donations to the Anatomical Gift Program of the Jacobs School of Medicine and Biomedical Sciences at the University at Buffalo (UB). The CT data were collected at the Center for Biomedical Imaging of the UB Clinical and Translational Science Institute (CTSI). The UB Anatomical Gift Program and the UB Department of Pathology and Anatomical Sciences approved of the use of these CT data in the current investigation.

This population consisted of both males and females, with an average age of 71.9 years and an age range of 45–92 years. The focus of the current study is the application of a new computational approach to evaluate potential differences in the regional geometric properties of healthy and osteoporotic femurs of humans in general. Given that the skeletal system is subject to changes associated with aging and differences due to sexual dimorphism, a limitation of the current study is that it only assesses osteoporosis in humans overall [30]. Future research is required in order to identify how osteoporosis influences the femur structure in males and females of specific age demographics.

The study consisted of a healthy (*n* = 31) experimental group and an osteoporosis (*n* = 11) experimental group. Several different methods are typically used to assess bone mass for the diagnosis of osteoporosis, and clinicians have been encouraged to diagnose osteoporosis in any older individual that exhibits high fracture risk to help address the underdiagnosis and undertreatment of this disease [31,32,33]. Most commonly, evaluation of fracture risk is carried out with a bone mineral density (BMD) test using dual-energy X-ray absorptiometry (DXA) in countries where this technology is widely available [34]. BMD tests using DXA were not available for use with the anatomical gifts in the current study. Therefore, osteoporosis was identified using the loss of bone mass in the femur structure extracted from the CT data, which were obtained using uniform Hounsfield units across all specimens with previously established procedures for extracting osteological tissue structure from the CT data [35,36].

The purpose of the current study is to examine the geometric properties of femurs with and without osteoporosis in general. Therefore, rare and unusually advanced cases of severe osteoporosis in which large portions of the femur are entirely absent are beyond the scope of the current investigation and not included within the osteoporosis experimental group. Consequently, the findings regarding the geometric property disparities between healthy and osteoporotic femurs may actually underestimate the morphological and biomechanical consequences of osteoporosis.

### 2.2. Ethics Approval

This study was performed in accordance with the ethical standards laid out in the 1964 Declaration of Helsinki and its later amendments or comparable ethical standards. The donor cadavers and the associated digital data are the property of the State University of New York at Buffalo. All informed consents were obtained from the donors prior to death by the University at Buffalo’s Anatomical Gift Program. Great care was taken in this study to ensure that all potentially identifiable digital cadaver data were removed to make all data anonymous.

### 2.3. 3D Model Generation

To ensure greater 3D model morphological accuracy and data validity, uniform segmentation was applied to every specimen in the study. This segmentation of osteological structures was carried out using the Kittler–Illingworth (KI) algorithm with robust intensity value thresholding [36,37,38]. All segmentation was completed using the open-source imaging platform 3D Slicer, and the data were exported as stereolithography (.stl) files [39]. Each of these exported femur structures consisted of both the external structure and the internal medullary cavity structure.

The 3D model optimization was completed using MeshLab, which is open-source 3D mesh processing software [40]. The 3D models were processed using automated functions for isolated piece removal, duplicate face removal, intersecting face removal, T-vertices removal, surface mesh hole closure, and non-manifold edge repair. A consistent mesh complexity of 20,000 triangular faces was used for all specimens in order to attain greater morphological accuracy for the 3D anatomical models [41]. Automated orientation of the 3D models was conducted in order to remove any potential error that would be attributed to rotational and translational differences between the specimens [40].

### 2.4. Automated Osteological Sampling

The femur specimen’s biomechanical length was measured in the publicly available software MeshMixer [42]. The biomechanical length was defined as the average of the measurements between the inferiormost aspect of the superior femoral neck and the distalmost point of medial condyle, as well as between the inferiormost aspect of the superior femoral neck and distalmost point of the lateral condyle, as defined in [43,44]. The y-z plane was defined as sagittal, and the x-z plane was defined as coronal. Automated sampling of each femur was used to obtain 60 evenly spaced cross-sections from the diaphysis ranging from 20 percent (distal) to 80 percent (proximal) of the biomechanical length, which was consistent with previous research [21,45,46,47].

The 3D anatomical models of the femurs were analyzed in the R programming language and software environment with the alphahull, Rvcg, colorRamps, raster, morphomap, and rgl libraries [21,48,49,50,51,52,53,54]. The periosteal and endosteal contours were automatically extracted from each cross-section. In all, 21 semilandmarks were obtained from each contour by sampling with equiangular radii originating from the medullary cavity centroid, which resulted in a total of 1260 semilandmarks per specimen [21]. These mathematically applied semilandmarks were utilized for analysis because diaphyseal bone is an extensive structure that contains very few reliable anatomical landmarks for the comparison of specimens [21,23,55,56,57].

### 2.5. Analysis

The cross-sections from each of the 3D models were used to evaluate the morphology of the healthy and pathological femur specimens. The periosteal and endosteal data from the diaphyseal region of the 3D anatomical models were also used to calculate several measurements. Cross-section area measurements were obtained for the total specimen cross-sectional area (i.e., the osteological structure as well as the medullary cavity) and medullary area. Also, the mean bone thickness, minimum bone thickness, and maximum bone thickness were measured at a given cross-section. In addition, periosteal perimeter and endosteal perimeter were calculated from each of the cross-sections.

The cross-sectional data were also used to calculate several parameters that are commonly used to evaluate the biomechanical attributes of osteological structures [23,58,59]. Beam theory, which is an established approach for quantifying long bone biomechanical properties, was applied to the diaphyseal region of the femur [20,28,60,61]. Specifically, the following formulae were utilized in the calculation of the biomechanical data consistent with [21].

The area moment of inertia around the x axis $\left(I_{x}\right)$ for a section was calculated as follows:

(1) $$I_{x}=\int y^{2}dA$$

The area moment of inertia around the y axis $\left(I_{y}\right)$ was defined as

(2) $$I_{y}=\int x^{2}dA$$

In addition, the minimum and maximum moments of inertia were calculated. The minimum area moment of inertia $\left(I_{min}\right)$ was determined using the equation

(3) $$I_{min}=\frac{1}{2}\left(I_{x}+I_{y}\right)-\frac{1}{2}\sqrt{{\left(I_{y}-I_{x}\right)}^{2}+{\left(4I_{xy}\right)}^{2}}$$

where

(4) $$I_{xy}=\int xydA$$

The calculation of the maximum area moment of inertia $\left(I_{max}\right)$ was carried out as follows:

(5) $$I_{max}=\frac{1}{2}\left(I_{x}+I_{y}\right)+\frac{1}{2}\sqrt{{\left(I_{y}-I_{x}\right)}^{2}+{\left(4I_{xy}\right)}^{2}}$$

Several section moduli were also calculated for each section. The maximum chord lengths from the x axis and y axis (i.e., $d_{y}$ and $d_{x}$) were used to find the moduli around the x and y axes. The section modulus about the x axis $\left(Z_{x}\right)$ was determined by the formula

(6) $$Z_{x}=\frac{I_{x}}{d_{y}}$$

while the section modulus about the y axis $\left(Z_{y}\right)$ was defined as

(7) $$Z_{y}=\frac{I_{y}}{d_{x}}$$

The minimum section modulus and maximum section modulus were calculated using the maximum chord lengths (i.e., $d_{y\theta }$ and $d_{x\theta }$) from the axes rotated by θ. The minimum section modulus $\left(Z_{min}\right)$ was determined with the formula

(8) $$Z_{min}=\frac{I_{min}}{d_{x\theta }}$$

Similarly, the maximum section modulus $\left(Z_{max}\right)$ was calculated as follows:

(9) $$Z_{max}=\frac{I_{max}}{d_{y\theta }}$$

Theta (θ), which describes the angle between the principal axis (maximum moment of inertia) or major axis and the x axis (mediolateral axis of the cross section) was calculated with the following equation:

(10) $$\theta =\frac{1}{2}\tan^{-1}\frac{2I_{xy}}{I_{y}-I_{x}}$$

The calculation of the polar section modulus $\left(Z_{pol}\right)$ was determined using the distance (*r*) from the centroid to the *dA* and the formula

(11) $$Z_{pol}=\frac{\int r^{2}dA}{r_{max}}$$

The polar moment of inertia (J) was determined with the equation

(12) $$J=\int \left(x^{2}+y^{2}\right)dA=\int x^{2}dA+\int y^{2}dA=I_{x}+I_{y}$$

where *x* is the distance from the y axis, *y* is the distance from the x axis, and *dA* refers to the discretized elements of the section.

Additionally, the cortical area (CA), a measurement often utilized as an indicator of the biomechanical properties [24,28,62], was calculated using the cross-section semilandmarks and the formula

(13) $$A=\frac{1}{2}|\sum_{i=1}^{n-1}x_{i}y_{i+1}+x_{n}y_{1}-\sum_{i=1}^{n-1}x_{i+1}y_{i}-x_{1}y_{n}|$$

## 3. Results

The data differed between the healthy femur experimental group and osteoporosis experimental group for several measurements (Figure S1). The total specimen cross-sectional area data exhibited values for the healthy femur experimental group that were greater than those of the osteoporosis experimental group at all sampling locations (i.e., 80 percent biomechanical length, 60 percent biomechanical length, 40 percent biomechanical length, and 20 percent biomechanical length). Analysis of these total area data revealed that these differences between the healthy experimental group and the osteoporosis experimental group were statistically significant across most of the femur (Table S1). Differences in the medullary area data were statistically significant at the 60 percent biomechanical length and 40 percent biomechanical length sampling locations.

In general, measurements of the bone thickness displayed statistically significant differences between the experimental groups. The mean bone thickness of the healthy experimental group was greater than that of the osteoporosis experimental group at all of the sampling locations along the femur. Similarly, the minimum bone thickness data were greater for the healthy experimental group than for the osteoporosis experimental group at all sampling locations. The maximum bone thickness data were comparable to the mean thickness and minimum thickness data, with greater values occurring for the healthy femurs than for the osteoporotic femurs. These differences in maximum bone thickness were statistically significant at the 80 percent biomechanical length, 60 percent biomechanical length, and 40 percent biomechanical length locations but did not reach the threshold of statistical significance at the 20 percent biomechanical length sampling location.

**Figure 1:**
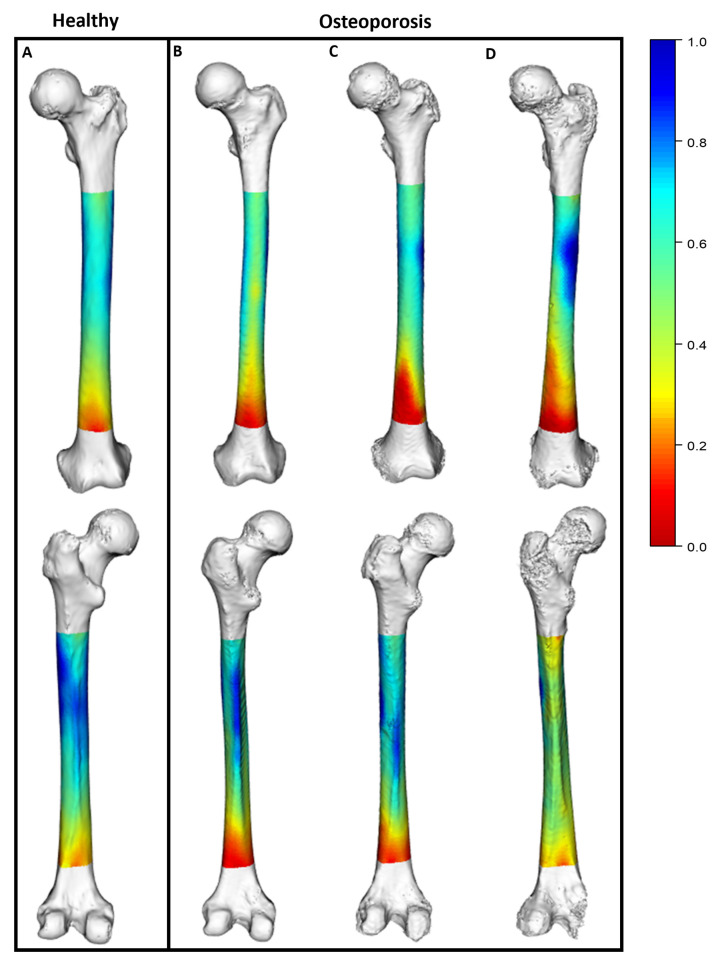
Three-dimensional models exhibiting disparities of morphological data between healthy and osteoporotic femurs. Heat maps show scaled bone thickness data corresponding with each location on the femur. (High thickness depicted in blue, ranging to low thickness shown in red). (**A**) Healthy femur. (**B**–**D**) Three examples of femurs with osteoporosis. Anterior view (above) and posterior view (below) are shown.

Although differences in the mean bone thickness were present at all of the sampling locations, the disparities in bone thickness between the healthy femurs and the osteoporotic femurs were not uniform across the diaphysis (Figure S2). Greater differences in mean bone thickness occurred between the 60 percent and 40 percent biomechanical lengths. In addition, the osteoporotic femurs exhibited a disproportionate reduction in bone thickness at the anterior and lateral regions of the distal diaphysis (20–45 percent biomechanical length). Figure 1 shows how the healthy and osteoporotic femurs exhibited different patterns of bone thickness across the femur.

**Figure 2:**
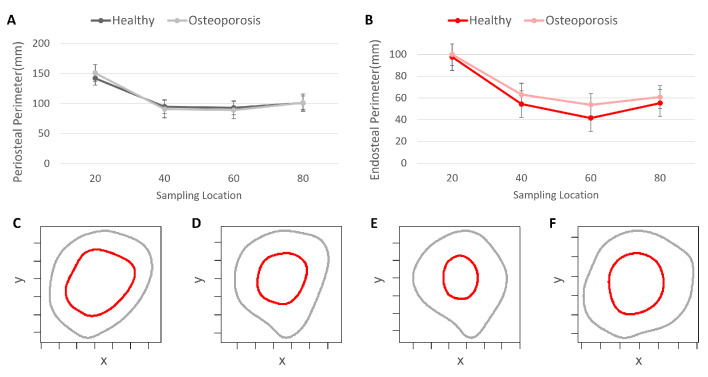
Perimeter data from the healthy and osteoporosis experimental groups. (**A**) Periosteal perimeter data. (**B**) Endosteal perimeter data. (**C**) Periosteal and endosteal perimeters at 80% biomechanical length (BL). (**D**) Periosteal and endosteal perimeters at 60% BL. (**E**) Periosteal and endosteal perimeters at 40% BL. (**F**) Periosteal and endosteal perimeters at 20% BL. Cross-section perimeter examples from a healthy femur.

The periosteal perimeter data exhibited the greatest consistency between the healthy and osteoporosis experimental groups. No statistically significant differences in the periosteal perimeter were detected at any of the sampling locations along the femur (Figure 2). Alternatively, the endosteal perimeter data had statistically significant differences at the 80 percent biomechanical length, 60 percent biomechanical length, and 40 percent biomechanical length locations (Table S2).

**Figure 3:**
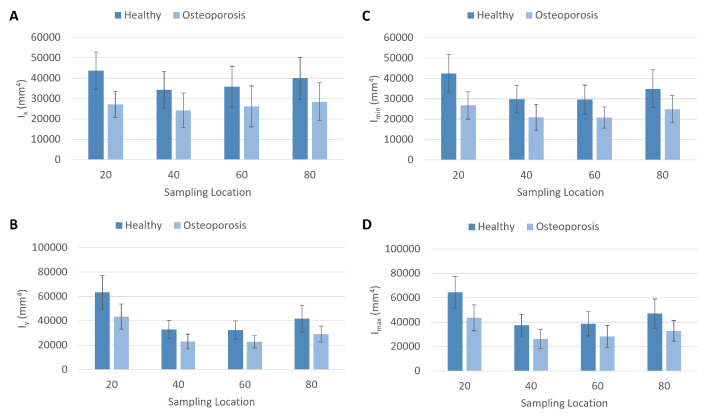
Plots of area moment of inertia data from the healthy and osteoporosis experimental groups. (**A**) Area moment of inertia around the x axis $\left(I_{x}\right)$. (**B**) Area moment of inertia around the y axis $\left(I_{y}\right)$. (**C**) Minimum area moment of inertia $\left(I_{min}\right)$. (**D**) Maximum area moment of inertia $\left(I_{max}\right)$. Sampling location = percentage of biomechanical length.

The area moment of inertia around the x axis and area moment of inertia around the y axis were greater in the healthy femurs than in the osteoporotic femurs at the 80 percent biomechanical length, 60 percent biomechanical length, 40 percent biomechanical length, and 20 percent biomechanical length locations (Figure 3). These differences between the experimental groups were statistically significant at all sampling locations along the femur (Table S3). Additionally, statistically significant differences occurred in the minimum area moment of inertia data and the maximum area moment of inertia data between the healthy experimental group and the osteoporosis experimental group. The average area moment of inertia data of the osteoporosis experimental group had values that were approximately 62–73 percent of those from the healthy experimental group. The greatest difference in all of these measurements was evident at the 20 percent biomechanical length location. At this most distal sampling location, the osteoporotic femur average area moment of inertia around the x axis was only 62 percent of the healthy femur average.

**Figure 4:**
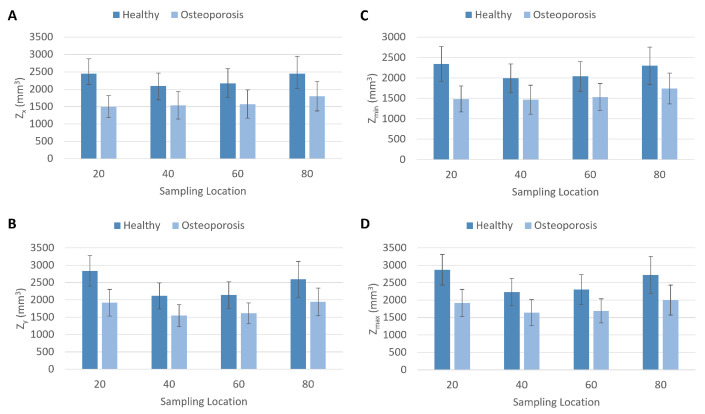
Plots of section modulus data from the healthy and osteoporosis experimental groups. (**A**) Section modulus about the x axis $\left(Z_{x}\right)$. (**B**) Section modulus about the y axis $\left(Z_{y}\right)$. (**C**) Minimum section modulus $\left(Z_{min}\right)$. (**D**) Maximum section modulus $\left(Z_{max}\right)$. Sampling location = percentage of biomechanical length.

The section modulus about the x axis data as well as the section modulus about the y axis data were greater for the healthy experimental group than for the osteoporosis experimental group, a finding that occurred at all sampling locations (Figure 4). In addition, the minimum section modulus and maximum section modulus values of the healthy experimental group were greater than the corresponding values of the osteoporosis experimental group. These dissimilarities in the data between the experimental groups were statistically significant at all of the sampling locations (Table S4). Overall, the section moduli values of the osteoporosis experimental group were approximately 61–76 percent of those in the healthy experimental group. The largest dissimilarities within each of the section modulus measurements occurred at the 20 percent biomechanical length location. Specifically, the section modulus about the x axis at the 20 percent biomechanical length location was the measurement that had the greatest disparity. The osteoporotic femurs had an average section modulus about the x axis that was merely 61 percent of the average section modulus about the x axis of the healthy femurs.

**Figure 5:**
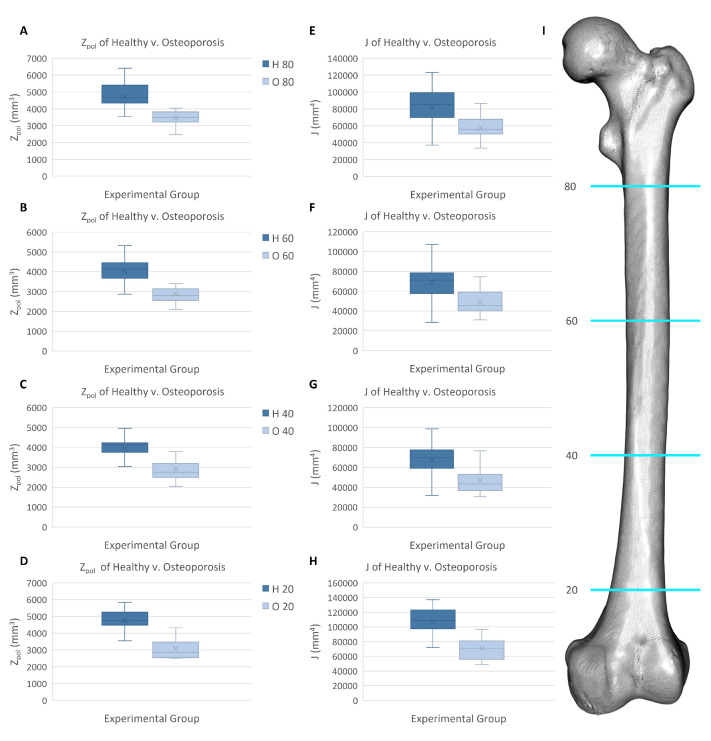
Biomechanical data of the healthy and osteoporosis experimental groups at four sampling locations along the biomechanical length (BL) of the femur. (**A**) Polar section modulus $\left(Z_{pol}\right)$ at 80% BL. (**B**) $Z_{pol}$ at 60% BL. (**C**) $Z_{pol}$ at 40% BL. (**D**) $Z_{pol}$ at 20% BL. (**E**) Polar moment of inertia (*J*) at 80% BL. (**F**) *J* at 60% BL. (**G**) *J* at 40% BL. (**H**) *J* at 20% BL. (**I**) Femur 3D model showing sampling locations (blue).

The healthy and osteoporosis experimental groups displayed statistically significant differences in the polar section modulus at sampling locations across the diaphysis (Table S5). At the 80 percent biomechanical length, 60 percent biomechanical length, and 40 percent biomechanical length locations, the osteoporotic femurs had average values that were about 72–74 percent of the corresponding values observed in the healthy femurs (Figure 5). The largest difference in the polar section modulus data occurred at the 20 percent biomechanical length location, where the average value for the osteoporotic femurs was approximately 65 percent of the average value for the healthy femurs. In contrast, the theta values remained consistent between the experimental groups at all sampling locations.

Akin to the polar section modulus findings, the polar moment of inertia data were significantly different between the healthy and osteoporosis experimental groups. The 80 percent biomechanical length, 60 percent biomechanical length, and 40 percent biomechanical length sampling locations displayed similar variation between the data of the experimental groups, with the osteoporosis experimental group having mean polar moment of inertia values that were approximately 70–72 percent of the healthy experimental group values. A more marked difference occurred at the 20 percent biomechanical length location, where the osteoporotic femurs had a mean value that was about 66 percent of the mean value found for the healthy femurs.

**Figure 6:**
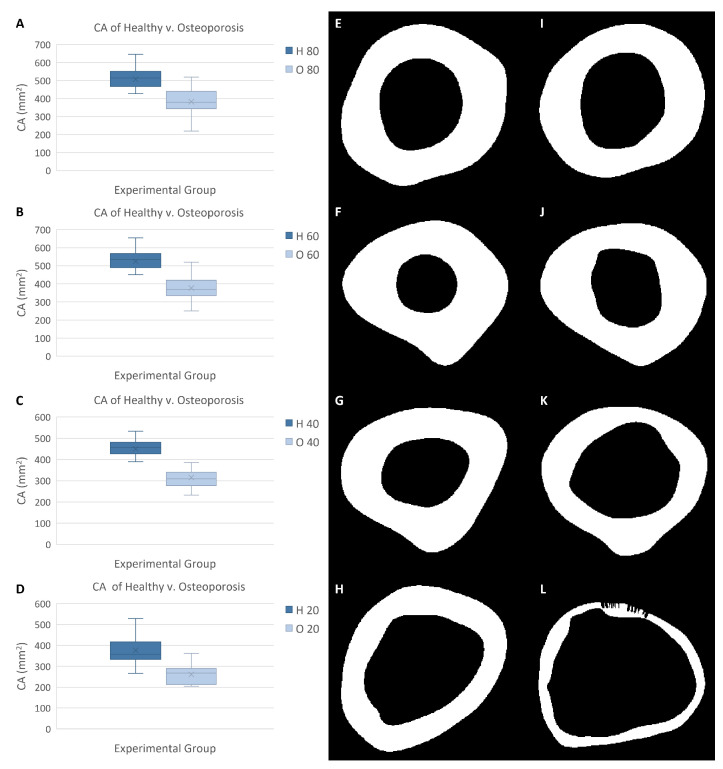
Cortical area (CA) data of healthy and osteoporosis experimental groups. (**A**) CA data from 80% biomechanical length (BL). (**B**) CA from 60% BL. (**C**) CA from 40% BL. (**D**) CA from 20% BL. (**E**) Healthy femur CA (shown in white) at 80% BL. (**F**) Healthy femur CA at 60% BL. (**G**) Healthy femur CA at 40% BL. (**H**) Healthy femur CA at 20% BL. (**I**) Osteoporotic femur CA at 80% BL. (**J**) Osteoporotic femur CA at 60% BL. (**K**) Osteoporotic femur CA at 40% BL. (**L**) Osteoporotic femur CA at 20% BL.

Similar to most of the other quantitative measurements, statistically significant differences in the cortical area data of the healthy and osteoporosis experimental groups were evident at all sampling locations. In all cases, the healthy experimental group had cortical area data that were greater than those of the osteoporosis experimental group (Figure 6). The osteoporotic femurs had a mean cortical area that was approximately 75 percent of the mean cortical area of the healthy femurs at the 80 percent biomechanical length location. Greater differences occurred at the 60 percent biomechanical length, 40 percent biomechanical length, and 20 percent biomechanical length sampling locations. The osteoporotic femurs had cortical areas that were 72 percent, 70 percent, and 70 percent of the cortical areas of the healthy femurs, respectively.

## 4. Discussion

The current study utilized 3D anatomical models to comprehensively examine the geometric data of healthy femurs and those with osteoporosis. Although these data represent both the morphological attributes and biomechanical attributes of the bones based on beam theory, it is important to note that bone strength is also dependent on the material properties of the specimen being tested [20,28,60,61]. The ongoing study of osteoporosis should continue to examine how variations in the osteological tissue material properties influence the biomechanical performance of various bones overall and at localized regions along bones.

Multiple measurements of morphological data from the healthy femur experimental group were consistent with previous studies that focused on healthy femurs. Specifically, the total area and medullary area data were comparable to those found in prior research [21,63]. Additionally, the healthy femur cortical area, section modulus about the x axis, section modulus about the y axis, and polar section modulus data were similar to those in previous examinations of healthy femurs [24,63].

Statistically significant differences between the healthy femurs and osteoporotic femurs were evident in multiple measurements at several locations across the diaphysis. These results are consistent with previous research, which found that the geometric properties measured from diaphyseal bone are effective for detecting the loss of bone tissue that is due to osteoporosis [58]. In particular, the morphological differences between the femurs of the healthy and osteoporosis experimental groups were especially apparent in the mean bone thickness, minimum bone thickness, and maximum bone thickness, which exhibited statistically significant differences at nearly every sampling location along the femur.

What is more, the data indicate that these differences in femur morphology for the healthy and osteoporosis experimental groups were not uniform across the diaphysis. Compared with the healthy femurs, the osteoporotic femurs had a severely reduced bone thickness at the 40 percent biomechanical length to 60 percent biomechanical length locations, which was further supported by the osteoporotic femurs having significantly greater medullary areas at these sampling locations. Additionally, the results revealed that a disproportionate reduction in mean bone thickness occurred at the anterior and lateral regions of the distal diaphysis in the femurs with osteoporosis. These results for the femoral diaphysis are consistent with previous findings for the femoral neck, which showed localized reductions in cortical bone thickness that were associated with fractures [14].

The geometric properties of the moments of inertia and section moduli are considered to be among the best data for assessing biomechanical capacities [59]. Compared with the healthy femur data, the osteoporotic femur data exhibited the greatest relative decreases in the moments of inertia and section moduli at the 20 percent biomechanical length location. Specifically, the greatest relative differences between the healthy and osteoporotic femurs occurred in the area moment of inertia about the x axis and the section modulus about the x axis. These results demonstrate that the osteoporotic femurs, when compared with the healthy femurs, were proportionately less resistant to bending in the anteroposterior plane at the distal diaphysis (i.e., 20 percent biomechanical length), a finding which suggests that osteoporotic femurs may be particularly vulnerable to fractures in this part of the femur [45,64].

The results also suggest that torsional rigidity is reduced in osteoporotic femurs when compared with healthy femurs. The polar section modulus and the polar moment of inertia data exhibited statistically significant differences at all sampling locations. For each measurement, the greatest disparity between the healthy and osteoporotic femurs was found at the 20 percent biomechanical length location, which suggests that osteoporotic femurs might be particularly less resistant to torsional forces at the distal diaphysis.

The cortical area data, which are indicative of the rigidity or strength of the femur when it comes to axial compression or compressive loading, were also significantly smaller at all sampling locations for the osteoporotic femurs when compared with the healthy femurs [24,28,45,64,65,66,67,68]. Interestingly, unlike other measurements, the cortical area data showed the greatest differences between the healthy and osteoporotic femurs at the 20 percent and 40 percent biomechanical length locations. Therefore, it appears that femurs afflicted with osteoporosis may be more susceptible to compressive fractures in the inferior half of the diaphysis. Intriguingly, these findings are consistent with patient outcomes in which diaphyseal and distal femur fractures exhibit poor healing (i.e., fixation failure typically due to nonunion), in which reoperation is necessary for 13.4 percent and 6.1 percent of patients, respectively [69].

**Figure 7:**
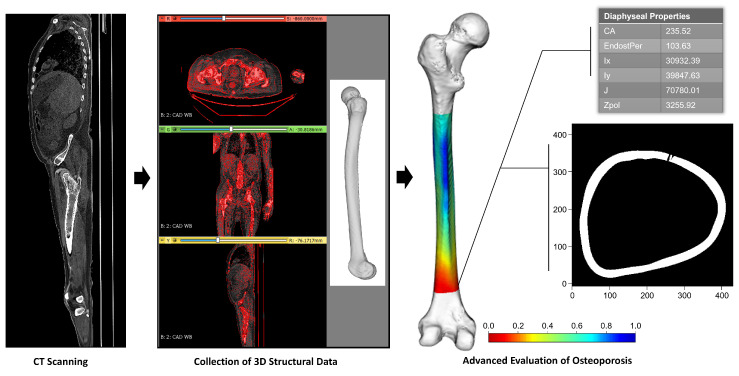
Conceptualization of how CT scans could be used to obtain comprehensive, patient-specific information about osteoporosis risk. Left femur with heat map showing regional bone thickness, a sampling location showing a cross-section of osteoporotic bone, and an example of regional quantitative data that can be calculated at a given sampling location. Cortical area (CA), endosteal perimeter (EndostPer), area moment of inertia around the x axis $\left(I_{x}\right)$, area moment of inertia around the y axis $\left(I_{y}\right)$, polar moment of inertia (*J*), and polar section modulus $\left(Z_{pol}\right)$.

Computational evaluation of osteoporosis may be helpful for reducing the high levels of mortality, the high levels of disability, and the great burden on the healthcare system caused by osteoporosis-associated femur fractures [1,9,10,11]. Such an approach could use patient CT data to offer comprehensive information about the condition of a patient’s bones, including the status of osteoporosis at specific regions along a bone with precise quantitative morphological and biomechanical data from regions of interest (Figure 7). This type of advanced evaluation could offer patient-specific insights about regions of bone that are especially vulnerable to fracturing. This information could be used to inform a variety of advanced preventive treatments.

## 5. Conclusions

Because of the high risk of death associated with femur fractures and the fact that osteoporosis-associated fractures are an unsolved problem in orthopedic surgery, it is essential to fully understand how osteoporosis alters the morphological and biomechanical characteristics of the femur [9,10,12]. The results support the hypothesis that healthy and osteoporotic femurs exhibit localized disparities in their geometric properties rather than uniform differences in these properties across the diaphysis. Furthermore, the results demonstrate that the 3D models obtained from CT scans can be used to identify specific, quantifiable differences in the shape and associated biomechanical properties of osteoporotic femurs. This strategy could be used to improve diagnostic procedures for detecting high fracture risk, to inform novel patient-specific injury prevention treatments, and to develop advanced surgical solutions for treating femur fractures.

## Data Availability

The data are contained within the article or Supplementary Materials.

## Supplementary Materials

The following supporting information can be downloaded at: https://www.mdpi.com/article/10.3390/jpm13020321/s1.

## Abbreviations

The following abbreviations are used in this manuscript:

CT	Computed tomography
UB	Universtity at Buffalo
CTSI	Clinical and Translational Science Institute
KI	Kittler–Illingworth
$I_{x}$	Area moment of inertia around the x axis
$I_{y}$	Area moment of inertia around the y axis
$I_{min}$	Minimum area moment of inertia
$I_{max}$	Maximum area moment of inertia
$Z_{x}$	Section modulus about the x axis
$Z_{y}$	Section modulus about the y axis
$Z_{min}$	Minimum section modulus
$Z_{max}$	Maximum section modulus
θ	Theta
$Z_{pol}$	Polar section modulus
J	Polar moment of inertia
CA	Cortical area
BL	Biomechanical length
