# Supplementary material for: Advancing Osteoporosis Evaluation Procedures: Detailed Computational Analysis of Regional Structural Vulnerabilities in Osteoporotic Bone
Source: J Pers Med. 2023 Feb 13;13(2):321. doi: 10.3390/jpm13020321 (PMC9962183; doi:10.3390/jpm13020321)
Supplement: Supplementary file 1 [file jpm-13-00321-s001.zip › jpm-2178270-supplementary.pdf]

Supplementary Material:

Advancing osteoporosis evaluation procedures: Detailed computational analysis of regional structural vulnerabilities in osteoporotic bone

Matthew A. Wysocki and Scott T. Doyle

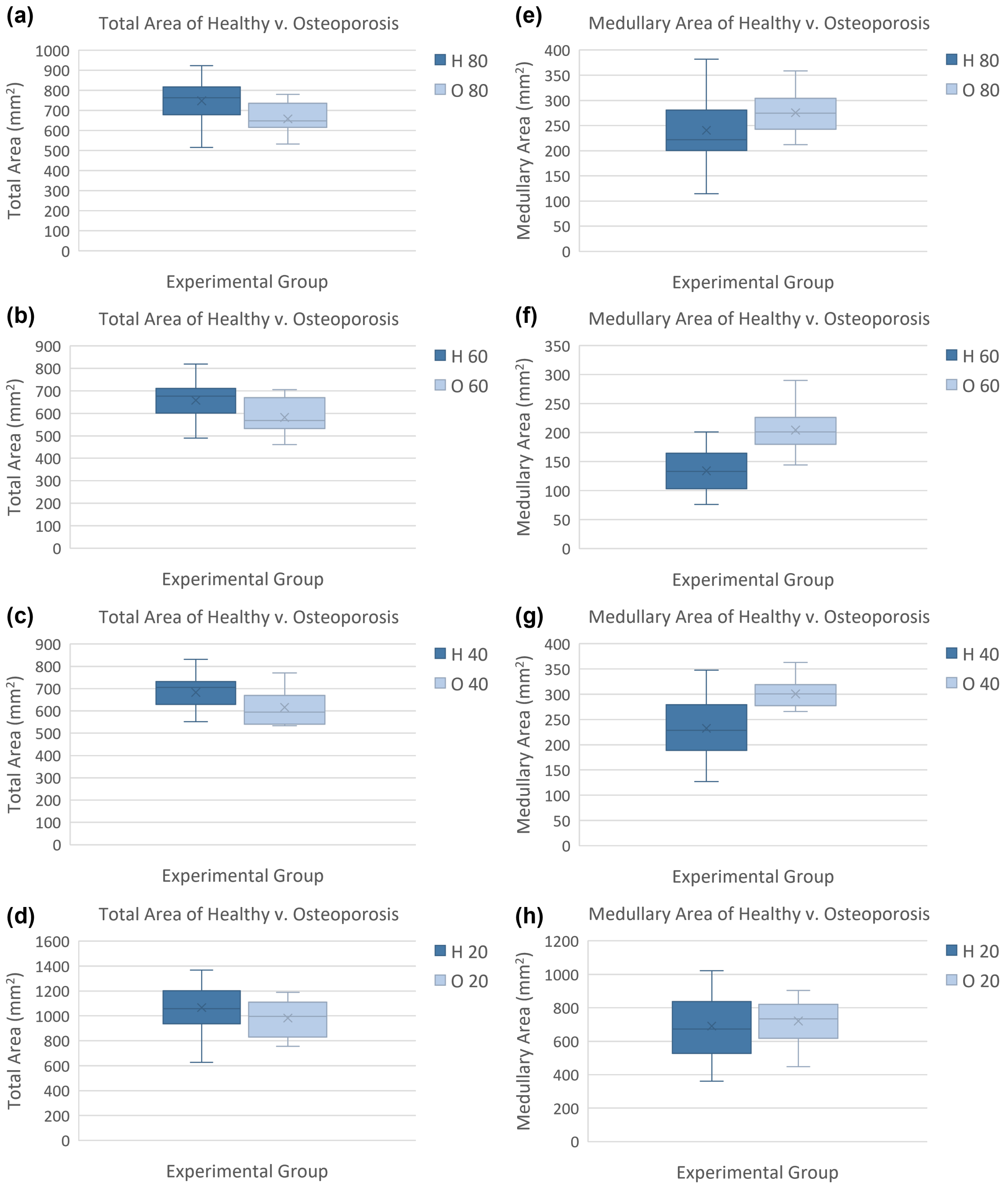

Figure S1. Total specimen cross-sectional area and medullary area of healthy and osteoporosis experimental groups. (A) Total area data at 80% biomechanical length (BL). (B) Total area data at 60% BL. (C) Total area data at 40% BL. (D) Total area data at 20% BL. (E) Medullary area data at 80% BL. (F) Medullary area data at 60% BL. (G) Medullary area data at 40% BL. (H) Medullary area data at 20% BL.

Table S1. Summary of t-test results from analyses of total specimen cross sectional area (Total A), medullary area (Medul A), mean bone thickness (Mean BT), minimum bone thickness (Min BT), and maximum bone thickness (Max BT) at the sampling locations along the femurs of the healthy experimental group and the osteoporosis experimental group. p-values shown; statistical significance indicated in bold.

	Total A	Medul A	Mean BT	Min BT	Max BT
80%	**1.10E-02**	1.05E-01	**2.43E-05**	**5.27E-04**	**3.03E-04**
60%	**1.09E-02**	**1.51E-06**	**7.33E-09**	**6.02E-07**	**6.39E-06**
40%	**3.26E-02**	**4.73E-04**	1**.38E-09**	**7.18E-08**	**5.27E-06**
20%	1.68E-01	6.36E-01	**1.76E-02**	**3.11E-02**	7.82E-01

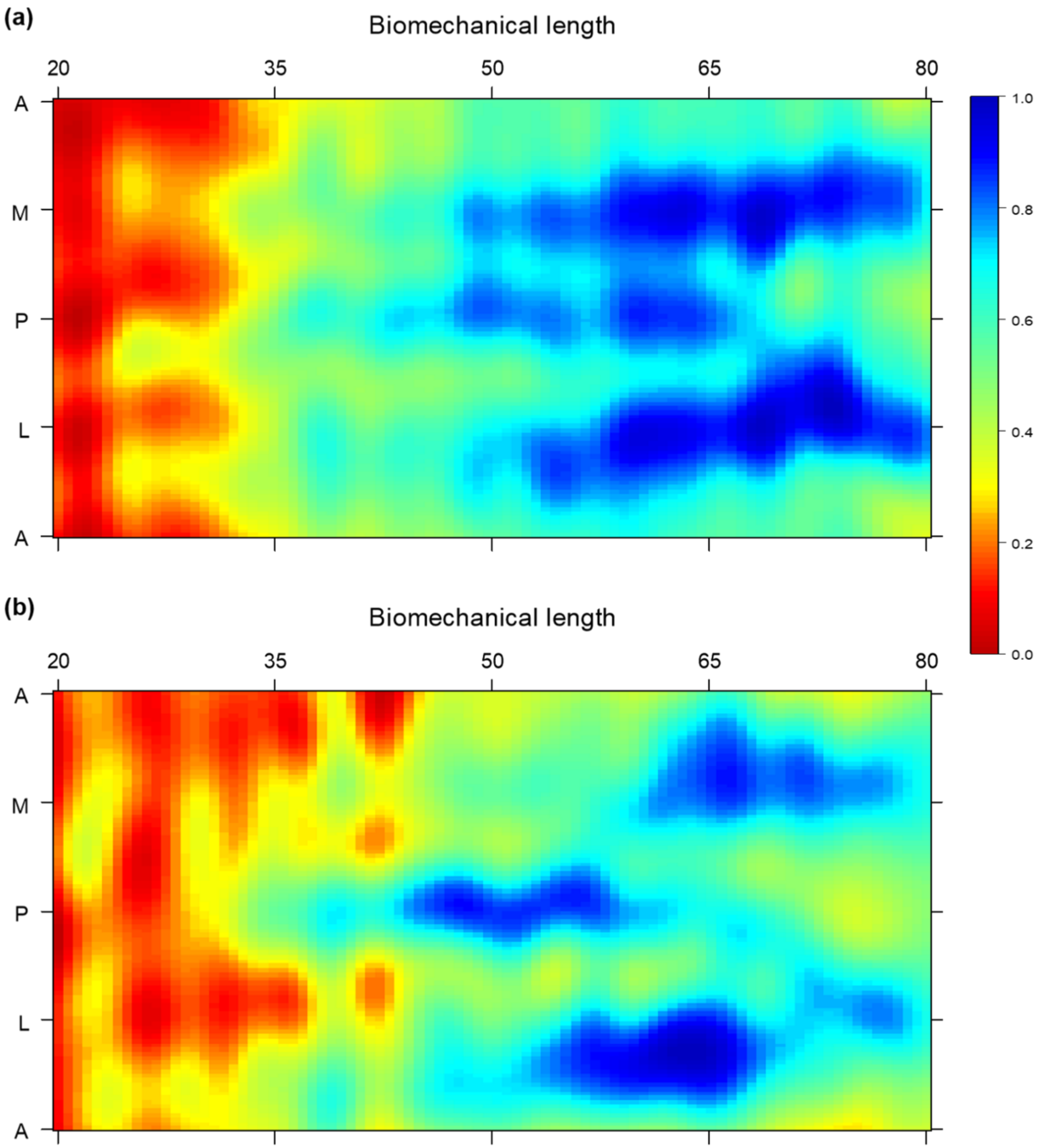

Figure S2. 2D plots showing the differences in bone thickness across the diaphyseal regions of healthy and osteoporotic femurs. a) Data from a healthy femur specimen. b) Data from a femur specimen with osteoporosis. Bone thickness data are scaled. Warmer colors indicate thinner bone. Biomechanical length = percentage of specimen biomechanical length; 20% = distal, 80% = proximal. A = anterior, M = medial, P = posterior, L= lateral.

Table S2. Summary of t-test results from analyses of the perimeter data from the sampling locations along the femurs of the healthy experimental group and the osteoporosis experimental group. p-values shown; statistical significance indicated in bold.

	Periosteal	Endosteal
80%	9.53E-01	**3.36E-02**
60%	8.88E-02	**9.47E-07**
40%	9.27E-02	**2.81E-04**
20%	3.62E-01	5.90E-01

Table S3. Summary of t-test results from analyses of area moment of inertia around the x axis (Ix), area moment of inertia around the y axis (Iy), minimum area moment of inertia (Imin), and maximum area moment of inertia (Imax) at the 4 sampling locations. p-values shown; statistical significance indicated in bold.

	Ix	Iy	Imin	Imax
80%	**2.81E-03**	**1.13E-03**	**2.56E-03**	**1.05E-03**
60%	**1.03E-02**	**4.02E-04**	**6.94E-04**	**4.58E-03**
40%	**3.02E-03**	**4.32E-04**	**5.99E-04**	**1.00E-03**
20%	**2.66E-06**	**1.06E-04**	**9.06E-06**	**3.43E-05**

Table S4. Summary of t-test results from analyses of section modulus about the x axis (Zx), section modulus about the y axis (Zy), minimum section modulus (Zmin), and maximum section modulus (Zmax) at the 4 sampling locations. p-values shown; statistical significance indicated in bold.

	Zx	Zy	Zmin	Zmax
80%	**5.39E-04**	**6.14E-04**	**9.96E-04**	**2.74E-04**
60%	**3.74E-04**	**2.83E-04**	**2.80E-04**	**1.60E-04**
40%	**2.20E-04**	**8.03E-05**	**1.75E-04**	**1.32E-04**
20%	**4.14E-08**	**4.78E-07**	**6.21E-07**	**2.26E-07**

Table S5. Summary of t-test results from analyses of polar section modulus (Zpol), polar moment of inertia (J), theta, and cortical area (CA) at the 4 sampling locations. p-values shown; statistical significance indicated in bold.

	Zpol	J	theta	CA
80%	**2.66E-04**	**1.02E-03**	4.62E-01	**3.18E-05**
60%	**5.37E-05**	**1.54E-03**	9.22E-01	**1.26E-06**
40%	**1.07E-04**	**6.73E-04**	5.06E-01	**1.34E-07**
20%	**1.87E-07**	**1.15E-05**	1.31E-01	**3.56E-06**
